# Socio-Economic Status and Language Development in Hearing Loss: A Critical Appraisal

**DOI:** 10.3390/audiolres13010015

**Published:** 2023-02-14

**Authors:** Paris Binos, Theodora Papastefanou, George Psillas

**Affiliations:** 1 Department of Rehabilitation Sciences, Cyprus University of Technology, Limassol 3036, Cyprus; 2 1st Otolaryngology Department, School of Medicine, Faculty of Health Sciences, Aristotle University of Thessaloniki, 541 24 Thessaloniki, Greece

**Keywords:** hearing loss, aural habilitation, language development, spoken language, language input

## Abstract

The impact of language input on children’s speech, language, and brain development was borne out of Hart and Risley’s famous “30-million-word gap”. A perspective bolstered by many studies in the last decade relates higher socio-economic status (SES) to better qualitative and quantitative differences in children’s speech. The logic chains found in these studies suggest that literacy development depends on language and brain development. Thus, brain building develops based on environmental experience and language input depends on the brain’s perception of the auditory information. This essay uses the latest published peer-reviewed research to outline the current landscape of the role of SES in the development of speech and language skills among children with hearing loss (HL) who are enrolled in auditory-driven habilitation programs. This essay argues that low SES families may provide sufficient input for their children. The outcome of auditory-driven programs implemented by speech-language pathologists (SLPs) seems to be detached from SES. The role of SES on this developmental trajectory remains unclear, and clinical practice may be related to other validated and robust parameters related to hearing loss.

## 1. Introduction

Evidence-based practice (EBP) is a process that has been developed to support speech–language pathologists (SLPs) making clinical decisions and to provide up-to-date clinical practice [1]. SLPs are evidence-based practitioners [2]. According to The American Speech-Language-Hearing Association (ASHA), “speech-language pathologists incorporate the principles of evidence-based practice in clinical decision making to provide high quality clinical care” [3]. The basic format for sharing new information in EBP is a short essay entitled critically appraised topic (CAT) which focuses on a clinical question. A CAT summarizes the evidence around a vital research question affecting clinical practice and is an important element of the evidence-based approach in the field of speech and language pathology (SLP). The CAT is a key step in the process since it is assessing the outcome of recent evidence and judge its value and relevance in a particular issue that is supported by relevant references. Though it is not the aim of this essay to signify all the factors contributing to the development of critical appraisal skills for clinicians, it is, however, crucial in mentioning their importance.

In the following introduction, we discuss the ongoing debate on the magnitude of socio-economic status (SES) in hard-of-hearing (HoH) children’s language skills when they participate in auditory-driven therapies. Today, the number of children under the age of five with permanent hearing loss in the UK alone is estimated to be 7200 and around 90% of deaf children are born to hearing parents [4,5,6,7]. Thus, the problem with hearing loss is that it keeps sound from reaching the brain, and the purpose of a hearing aid or cochlear implant (CI) is to stimulate and grow auditory neural connections of the brain as the cornerstone for spoken language and academics [8,9]. A cochlear implant is a device that can help children with severe to profound hearing loss acquire the ability to develop speech and language skills similar to normally hearing peers.

According to Greenwell and Walsh [10], there is a recorded interdisciplinary gap of training specific to article appraisal since clinicians can find it fascinating to depend upon research investigators’ interpretations. Over the past few years, evidence-based practice has been emphasized in all aspects of SLP. Extensive research has indicated that the early language environment plays a critical role in shaping future speech, language, and cognitive skills [1]. However, research must shed light on the factors that shape the quantitative and qualitative features of the early language environment, as well as the role of socio-economic status (SES), in the future linguistic competence and academic success of hard-of-hearing children.

Socio-economic status is not a narrow and simple factor, but a reflection of the economic and social background of a household. Many other studies explore this factor under the prism of parental education or income alone, but the aim of this essay is to reveal the role of SES in the development of speech and language skills among children with hearing loss (HL) enrolled in auditory-driven habilitation programs. How do HoH children learn to listen, talk, and read like children with typical hearing? There is a “logic chain” that SLPs can take between basic brain biology and how children can talk and read. This “logic chain” includes different pieces needed to develop a child’s brain and each component is a link in this chain. Brain development is the first piece of the chain and it is directly affected by the language input the child receives through his/her hearing aid. Hearing technology, language development, listening and spoken language (LSL), early intervention, and literacy development are the components of this “logic chain” where each piece serves as a critical link in the chain [11]. Children’s earliest experiences in life are foundational to brain development [12]. This CAT examined the role of socio-economic status (SES) as a key factor in developing speech and language skills among children with hearing loss (HL) who are enrolled in auditory-driven programs.

The quality of language exposure has frequently been related to a family’s socio-economic status (SES). In language acquisition literature, SES has been operationalized as parental education (most often maternal education), household affluence (estimated from parental occupation, entitlement to free school meals, or estimated from postcodes), or indices of deprivation. Most studies have employed a single measure of SES, although composite measures have been claimed to be more valuable because they capture multiple components of the child’s environment. The impact of qualitative components of language exposure is now the focus of a growing amount of research [13,14].

Today, the habilitation of HoH children is based mainly on EBP using auditory-driven models, such as auditory verbal therapy (AVT), or aural/oral approaches that combine speech reading [15]. A child develops spoken language through residual hearing and optimal amplification. These therapies take advantage of neural plasticity to ”build” the brain. The role of caregivers is upgraded and differs from that of traditional speech–language therapy (SLT).

In Hart and Risley’s [16] landmark study from typically developing studies, the quantity of caregiver talk was established as the best predictor of future child linguistic outcomes. They identified an enormous difference in the quality and amount of language experience between participants at different levels of the socio-economic ladder. One-hour monthly recordings from the child’s first to third birthdays showed that the number of spoken words was significantly correlated with SES. Their estimation suggested that children are exposed to about 2500 words per hour while awake. According to The Outcomes of Children with Hearing Loss (OCHL team), if a child uses the hearing aid for an average of 8 h per day rather than throughout an entire 14-h walking day, she/he will hear 22 million fewer words during the first 4 years of life [17,18]. Thus, Hart and Risley [16] estimated that participants with high SES would have heard three times more child-directed speech than children with low SES. Their home-based findings, commonly referred to today as the “30-million-word gap”, concluded that children aged four years who came from the most advantaged backgrounds had heard 30 million more words than the least advantaged participants (45 versus 15 million words, respectively).

The influence of this study is substantial. Consequently, more studies influenced by this conclusion argue for quantitative and qualitative differences in the speech of diverse SES contexts [19,20]. Other studies have shown a significant link between caregiver involvement and children’s outcomes [21,22,23], but the role of SES remains unclear. Although significant disparities in language input are associated with family socio-economic status [24], other research regarding auditory-driven therapies, such as auditory–verbal therapy (AVT), has proven otherwise [25]. Socio-economic status did not play a role in spoken language outcomes only when the appropriate habilitation was provided [25]. The results revealed that even participants with low SES achieved significant language outcomes after the implementation of AVT, compared to those of their peers [26].

Similar outcomes have been reported in the literature regarding hearing loss. Thus, in their extensive systematic review from 1993 to 2015, Kaipa and Danser [26] mention that SES did not significantly influence the outcomes of AVT. Although SES plays a role in future speech and language outcomes, this is not the case at any time or in any case. Therefore, under specific conditions, such as when children receive an evidence-based therapy like AVT, an intense and highly structured habilitation, the role of SES in communication skills is undermined. The conclusions of this study are as follows: There was no effect of SES on linguistic skills among children with hearing loss if they were part of an AVT habilitation. Another systematic review and meta-analysis explored the role of family environment in the language development of children with cochlear implants [26]. There was no statistical evidence for a link between SES and children’s language development. The meta-analysis resulted in an overall low and non-significant average correlation coefficient (r = 0.117, *p* = 0.262, 95% cochlear implant-CI = −0.087 to 0.312). In a recent meta-analysis of 22 studies, Piot et al. [27] used an innovative technology consisting of automated analyses of daylong recordings, called Language Environment Analysis (LENA) [28], and followed the PRISMA guidelines for systematic reviews and meta-analyses. The authors provide a systematic way to measure the magnitude of SES in children’s language experiences. With an effect size of rz = 0.186, they found only a slight effect of SES on children’s language experiences.

Several studies have investigated SES and insurance type in relation to children’s referral age and/or access to therapy [28,29,30,31]. For example, Chang et al. [29] (49% of sample with Medicaid) found no association of insurance type with age at referral in Ohio, while Lester et al. [30] (64% of sample with Medicaid) found that private insurance was associated with earlier cochlear implantation (CI) in Washington, DC, and Iowa, respectively. Furthermore, Ramírez-Esperza et al. [32] studied the effect of language input on language development, comparing “parentese” speech to everyday speech. One of the parameters tested was SES role in these relationships. Although they found a strong relationship between the quality of speech input and the child, they did not find similar outcomes for the raw quantity. Moreover, Huttenlocher et al. [20] concluded similarly that parent input mediates the effects of SES on child language growth.

There is a recorded variability in linguistic input quantity and quality components since these significant disparities are partially or not all associated with family socio-economic status. The “anti-gap” side is enriched with the outcomes of a recent study by Romeo et al. [24], where language experience was measured based on real-world audio recordings of 36 SES diverse 4- to 6-year-old children. As a multivariate feature of several correlated factors, SES disparities are strongly related to environmental and neural factors. Thus, language input is crucial through qualitative measurements, rather than qualitative measurements. Conversational turns evoked greater left inferior frontal activation independent of SES. Dailey and Bergelson [33], in their recent quantitative meta-analysis of SES in language input to young children, have reported a minimal effect of SES on child-directed speech compared to the “30-million-word gap”. SES can be described as a limiting factor affecting linguistic input, even for families of lower SES [34].

## 2. Methods

### 2.1. Search Strategy and Clinical Question of This Essay

Key terms and their synonyms were developed from PICO elements (patient-intervention/assessment-comparison-outcome). The PubMed/MEDLINE database was searched in October 2022 and is presented in Appendix A. There are details provided with its terms of keywords and limits in the database in Appendix A. This essay, based on up-to-date relevant references, argues on an ongoing debate exploring the answer to a focused clinical question, “Is SES a crucial factor significantly related to speech and language skills among children enrolled in aural/oral programmes?”.

### 2.2. Limitations

The search was based on the PubMed/MEDLINE database, published between 2004 and May 2022. No other database was used. All research designs were not considered for inclusion if they were not peer-reviewed and published in English. Opinions, purely theoretical studies, systematic reviews, and meta-analyses were excluded (Table 1).

The search revealed a lack of study under the keywords, “*SES AND speech production AND hearing loss,*” since there were only four studies [35,36,37,38] related to humans. Finally, only two [35,38] remained because the other two were not directly related to the present research question and were not formed by PICO elements (see Table 2). SES is defined either as the rate of accessibility to hearing rehabilitation and impact on speech and language outcomes in hearing loss [35], or the access to therapy services between rural and urban children [38].

### 2.3. PICO

The PICO question formed based on the table below.

**Table 2:** PICO elements derived from the focused clinical question.

PICO elements	Well-built question by PICO element
P-Patient Group	Infants and Children with Hearing Loss
I-Intervention/Assessment	Auditory-driven approaches
C-Comparison	SES among typically developing children (TD) and Hard-of-Hearing
O-Outcome	The role of SES is dubious

## 3. Results

Smith et al. [35] recruited 600 patients from three tertiary-care academic centers between 2010 and 2012 by randomly selecting 3679 patients. Participants were separated into two groups based on SES (private insurance vs. Medicaid). The primary outcome measures were SLP evaluation and participation. This study aimed to evaluate the effects of socio-economic status (SES) on access to hearing rehabilitation, speech, and language therapy, and outcomes in children with hearing loss. This study was a Retrospective Chart Review. The results demonstrated that the pure tone average (PTA) did not differ between the two groups. There were no significant differences in the presence of speech delay (*p* = 0.62), receipt of SLP (*p* = 0.49), or speech language outcomes between the two groups (*p* = 0.45). Some of the limitations of this study were the potential bias introduced by the retrospective nature of the study, uncertainty regarding the relationship between insurance status and SES, simplified and preliminary SLP outcomes, and heterogeneity of data between institutions. The authors concluded that, despite the lower SES in children with HL, Medicaid allows equivalent access to rehabilitation and SLP, thereby achieving similar speech and language outcomes.

Noblitt et al. [38] recruited 35 parents of children who underwent CI (1996–2013) at a tertiary medical center. Twenty-one parents from rural and fourteen parents from urban residents completed the Parents’ Evaluation of Aural/Oral Performance of Children (PEACH) scores. This study aimed to assess the barriers of rehabilitation care for pediatric cochlear implant recipients. The authors used a cross-sectional questionnaire designed using PEACH. The results showed that low SES and Medicaid insurance were associated with a lack of local SLPs and medical CI complications. Limitations: Low SES was related to the delayed receipt of CI rehabilitation services. Low SES may impact a recipient’s language development. Some of the limitations of this study were the low response rate, bias in the results associated with the questionnaire study design, lack of generalizable outcomes regarding low SES populations, lack of mixed methodology that includes quantitative and qualitative data collection, use of a non-validated survey, speech outcomes were not feasible, and variability of rehabilitation services. Finally, the authors argued that low SES or Medicaid insurance were not significantly associated with PEACH scores but could affect hearing rehabilitation.

## 4. Discussion

In this essay, a CAT format was used to investigate the role of SES in the development of speech and language skills among auditory-driven children with HL. These results illustrate that low SES families may not provide sufficient input for their children. Moreover, the outcome of auditory-driven implementation by SLPs seems to be different from SES, and clinical practice may be related to other validated and robust parameters.

Smith et al. [35] argued that children with high or low SES did not differ significantly in terms of their performance. Similarly, Kaipa and Danser [26] demonstrated that SES did not significantly influence AVT outcomes. Although they recognized that SES plays a role in future speech and language outcomes, this was not the case at any time or in any case. Therefore, under specific conditions, such as when children receive evidence-based therapy like AVT, an intense and highly structured habilitation program, the role of SES in communication skills is undermined. The conclusions of this study are as follows: There was no effect of SES on linguistic skills among children with hearing loss if they were part of an AVT habilitation program. In contrast, Piot et al.’s [27] meta-analysis provided a systematic way to measure the magnitude of SES on children’s language experiences. With an effect size of rz = 0.186, the authors found only a slight effect of SES on the children’s language experience.

Noblitt et al. [38] stressed that low SES or Medicaid insurance was not significantly associated with PEACH scores but could affect hearing rehabilitation. This is in line with previous evidence suggesting that children in lower-income families are more likely to receive sign language teaching but less likely to report access to speech therapy [39]. However, there is a bulk of studies providing dubious results regarding the SES level, or the type of insurance and children’s referral age, or access to therapy [29,30,31]. Moreover, Sharma et al. [40] argued similar findings in their pragmatic, retrospective, and observational study. Participants wearing cochlear implants from different economic backgrounds with an annual income of less than $7500, between $7500 and $15,000, and even more than $15,000, performed equally well during their postoperative outcomes. Only the children of families with more than $15,000 scored SIR higher. They concluded that children with cochlear implants who followed the designated program of postoperative mapping and auditory verbal therapy for at least 1 year do equally well irrespective of the SES of the family. SES does not have a significant impact on the performance of children after cochlear implantation. Unfortunately, comprehensive tests for receptive or expressive language were not used for all the participants. In contrast, a study by Ozcebe et al. [41] linked the low SES, especially low levels of education in the family, to delays in cochlear implantation.

The early language development of typically developing children is quantitatively and qualitatively affected by their home environment, which may be correlated with SES [42,43]. This is the essence of the study by Hart and Risley [16]. This perspective of a “30-million-word gap” in language development between the “welfare” and the “professional” group received some statistical evidence during the years that parent input mediates the effects of SES on language development [16]. Nevertheless, the “gap” side does not remain intact.

Unfortunately, Hart and Risley’s [16] work included only family interactions involving the child and was conducted in the presence of an observer. Their study permitted observers to engage with the family and statistical differences were found only in two studies by Hart and Risley [16] and not in any other group. However, many studies have highlighted the positive influence of observers on family feedback [44,45]. New and robust research data suggest that there is no “massive word gap”. Sperry et al. [46] encouraged observers to interact with the family and they were added recordings from five different communities, two “working class”, two described as “poor”, and one “middle class”. At the same time, they assessed language input differently, including recordings as overheard speech and directed speech to the child. As Sperry et al. [46] stated, capturing only the speech addressed directly to children is insufficient. Community, rather than SES, influences parents’ input, and children from lower income households hear more speech than Hart and Risley [16] reported. These conclusions led Sperry et al. [46] to revisit Hart and Risley’s claims.

## 5. Conclusions

There are severe risks in supporting the views of Hart and Risley [16] despite their severe influence in today’s clinical practice that the HoH receives. However, claims supporting the idea that children from lower SES hear more speech than Hart and Risley’s claims are dubious. Language experience may matter, but it is now clear that not all aspects of language exposure do so equally. Recent studies reveal that child-directed speech quality is more helpful to infants and young children than quantity per se and parents’ vocabulary diversity at 30 months predicted children’s vocabulary at 42 months [47,48]. It is also clear that there is no massive word gap among the many populations. Future research may proceed beyond the “30-million-word gap” notion, including participants from a broader range of SES, cultural backgrounds, and databases. As Golinkoff et al. [42] state that there is a need for future research to include measurements of a high SES group, and proceed to the quality speech directed to children rather than overheard speech. The perspective that supports the notion that not all low SES families provide sufficient input for their children is groundless. Interestingly, children receiving Medicaid had equal access to hearing rehabilitation and achieved equal outcomes to privately insured children. Socio-economic status did not always play a key role in spoken language outcomes for the HoH children.

## Figures and Tables

**Table 1:** Data extraction of included studies.

Characteristics	Study 1 [35]	Study 2 [38]
Population	600 patients from 3 tertiary care academic centers, from 2010 to 2012 by random selection of a total of 3679 patients	35 Parents of children who received a CI (1996–2013) in a tertiary medical Center
Study Design	Participants were separated into two groups based on SES (private insurance vs. Medicaid). The primary outcome measures included SLP evaluation and Participation	21 parents from rural and 14 parents from urban residents completed PEACH scores
Aim	Evaluate the effect of socio-economic status (SES) on access to hearing rehabilitation and speech and language therapy and outcomes in children with hearing loss	Assess barriers to rehabilitation care for pediatric cochlear implant (CI) recipients
Methodology	Retrospective Chart Review	Cross-sectional questionnaire study using the Parent’s Evaluation of Aural/Oral Performance of Children (PEACH)
Outcomes	The pure tone average (PTA) did not differ between the two groups. There were no significant differences in the presence of speech delay (*p* = 0.62), the receipt of SLP (*p* = 0.49) or speech-language outcomes between the two groups (*p* = 0.45)	Low SES and Medicaid insurance were linked with a lack of local SLPs and medical CI complications. Low SES related to delay in receipt of CI rehab services. Low SES may impact the recipient’s language development.
Limitations	Potential bias introduced by the retrospective nature of the study Uncertainty about the relationship between insurance status and SES SLP outcomes were simplified and preliminary Heterogeneity of data between institutions	Low response rate Bias in the results associated with a questionnaire study design Lack of generalizable outcomes regarding low SES populations Lack of mixed methodology that includes quantitative and qualitative data collection Use of a non-validated survey Speech outcomes were not feasible Variability of rehab services
Conclusions	Despite lower SES in children with HL, Medicaid allows equivalent access to rehabilitation and SLP, achieving similar speech and language outcomes.	Low SES or Medicaid insurance were not significantly associated with PEACH scores but can affect hearing rehab.

## Data Availability

Not applicable.

## Appendix A. Keywords and Limits on Database

**Database**	**Keywords**	**Limits**
PubMed/Medline	(SES AND speech production AND hearing loss)	Humans English language Peer reviewed Evidence-based Reviews 2004–today
